# Differential, but not opponent, effects of l-DOPA and citalopram on action learning with reward and punishment

**DOI:** 10.1007/s00213-013-3313-4

**Published:** 2013-11-15

**Authors:** Marc Guitart-Masip, Marcos Economides, Quentin J. M. Huys, Michael J. Frank, Rumana Chowdhury, Emrah Duzel, Peter Dayan, Raymond J. Dolan

**Affiliations:** 1 Wellcome Trust Centre for Neuroimaging, Institute of Neurology, University College London, London, WC1N 3BG UK; 2 Ageing Research Centre, Karolinska Institute, SE-11330 Stockholm, Sweden; 3 Translational Neuromodeling Unit, Department of Biological Engineering, ETH and University of Zurich, Zürich, Switzerland; 4 Gatsby Computational Neuroscience Unit, University College London, London, W1CN 4AR UK; 5 Department of Cognition, Linguistics, and Psychological Sciences, Brown University, Providence, RI USA; 6 Institute of Cognitive Neuroscience, University College London, London, W1CN 4AR UK; 7 Institute of Cognitive Neurology and Dementia Research, Otto-von-Guericke-University, Leipziger Strasse 44, 39120 Magdeburg, Germany; 8 Department of Psychiatry, Psychotherapy and Psychosomatics, Hospital of Psychiatry, University of Zurich, Zürich, Switzerland

**Keywords:** Pavlovian, Instrumental, Reinforcement learning, Dopamine, Serotonin, Control

## Abstract

**Rationale:**

Decision-making involves two fundamental axes of control namely valence, spanning reward and punishment, and action, spanning invigoration and inhibition. We recently exploited a go/no-go task whose contingencies explicitly decouple valence and action to show that these axes are inextricably coupled during learning. *This results in a disadvantage in learning to go to avoid punishment and in learning to no*-*go to obtain a reward*. The neuromodulators dopamine and serotonin are likely to play a role in these asymmetries: Dopamine signals anticipation of future rewards and is also involved in an invigoration of motor responses leading to reward, but it also arbitrates between different forms of control. Conversely, serotonin is implicated in motor inhibition and punishment processing.

**Objective:**

To investigate the role of dopamine and serotonin in the interaction between action and valence during learning.

**Methods:**

We combined computational modeling with pharmacological manipulation in 90 healthy human volunteers, using levodopa and citalopram to affect dopamine and serotonin, respectively.

**Results:**

We found that, after administration of levodopa, action learning was less affected by outcome valence when compared with the placebo and citalopram groups. This highlights in this context a predominant effect of levodopa in controlling the balance between different forms of control. Citalopram had distinct effects, increasing participants’ tendency to perform active responses independent of outcome valence, consistent with a role in decreasing motor inhibition.

**Conclusions:**

Our findings highlight the rich complexities of the roles played by dopamine and serotonin during instrumental learning.

**Electronic supplementary material:**

The online version of this article (doi:10.1007/s00213-013-3313-4) contains supplementary material, which is available to authorized users.

## Introduction

The ultimate goal of behavioral control is to select policies that maximize reward and minimize punishment. To achieve this, animals are endowed with a flexible controller (typically referred to as instrumental) that learns choices on the basis of their contingent consequences. However, animals are endowed with an additional controller (called a Pavlovian controller) which produces stereotyped hard-wired behavioral responses to the occurrence of affectively important outcomes or learned predictions of those outcomes (Dickinson and Balleine 2002). Two central forms of Pavlovian control are active approach and engagement given the prospect of reward, and inhibition and withdrawal given the prospect of punishment (Gray and McNaughton 2000). Thus, in Pavlovian control, vigor and valence are coupled, and this could be a source of suboptimal behavior. Instrumental and Pavlovian controllers often prescribe the same policies in a manner that can accelerate the expression of good performance. These are the most common circumstances encountered by animals and humans alike. Everyone knows that obtaining a reward normally requires some sort of overt behavioral response (go to win), from picking berries in the forest, to buying them in a shop, or going to a restaurant to eat them. Similarly, the most efficient way to avoid a punishment is to avoid those actions that may lead to it (no-go to avoid losing); it’s better to keep off the road if you want avoid being driven over. However, when the Pavlovian and instrumental controllers are in opposition, behavioral output becomes suboptimal (Boureau and Dayan 2011; Breland and Breland 1961; Dayan et al. 2006). For example, if an unexpected car threatens a pedestrian while crossing the street, it is not uncommon that the pedestrian freezes (which is a highly suboptimal Pavlovian influence) before starting the appropriate running response (go to avoid losing). Similarly, a hunter will often need to remain completely still in the proximal presence of a potential prey (no-go to win), waiting for the optimal moment to act. Failure to be inactive during this critical period (another highly suboptimal Pavlovian influence) results in the prey escaping and the omission of the potential reward.

An important source of influence on the coupling between action and valence may arise from monoaminergic neuromodulation (Boureau and Dayan 2011; Cools et al. 2011; Gray and McNaughton 2000). Dopamine is believed to generate active motivated behavior (Berridge and Robinson 1998; Niv et al. 2007; Salamone et al. 2007) and to support instrumental learning (Daw and Doya 2006; Frank et al. 2004; Wickens et al. 2007) through model-free reward prediction errors (Bayer and Glimcher 2005; Morris et al. 2006; Schultz et al. 1997). These joint roles of dopamine on action invigoration and model-free reward prediction error signalling resonate with the involvement of dopamine in Pavlovian behaviors observed in experimental animals (Flagel et al. 2011; Parkinson et al. 1999). On the other hand, the role or the serotonergic system is more debated, but it appears closely related to behavioral inhibition in aversive contexts (Crockett et al. 2009; Dayan and Huys 2009; Soubrie 1986). In order to manipulate action and valence orthogonally, we and others have designed go/no-go tasks that involve four different conditions: go to win, go to avoid losing, no-go to win, no-go to avoid losing. These tasks have been used to show the involvement of serotonin in punishment-induced inhibition (Crockett et al. 2009) and dopamine in invigoration of actions that lead to reward (Guitart-Masip et al. 2012a).

However, the precise role played by these neuromodulators during learning has yet to be investigated. To explore these effects, we manipulated dopaminergic and serotoninergic systems during learning. Participants received placebo, levodopa, or citalopram. The pharmacological agents are assumed to affect postsynaptic levels of dopamine (Koller and Rueda 1998) and serotonin (Spinks and Spinks 2002), respectively. However, the balance of their influences on phasic and tonic aspects of these neuromodulators and the anatomical location of their sites of action are not clear. If the predominant effect *were to enhance* the coupling between action and valence typically associated with the Pavlovian control system, we would expect to see increased valence-specific Pavlovian interference on instrumental learning. Indeed, based on the bulk of the literature reviewed above, one would exactly expect that after levodopa administration. However, if the predominant effects of the drugs lay elsewhere, for instance, in the modulation of the contribution of prefrontal cortex to control (Hitchcott et al. 2007), then other effects might arise, such as a decrease in the extent of suboptimal behavior. Our results bear out the latter expectation. We found differential, but not opposing, roles for dopamine and serotonin on instrumental learning whereby boosting dopamine levels decreased the coupling between action and valence on the one hand, while boosting serotonin resulted in a valence-independent decrease in behavioral inhibition.

## Methods and materials

### Subjects

Ninety healthy volunteers were recruited from a subject pool associated with University College London’s Psychology Department and completed the pharmacological experiment. They received full written instructions and provided written consent in accordance with the provisions of University College London Research Ethics Committee. Participants were randomly assigned to one of three treatment groups: 30 participants received levodopa (13 female; age range, 17 years; mean, 24.07, SD = 4.08 years), 30 participants received citalopram (17 female; age range, 15 years; mean, 23.31, SD = 3.77 years), and 30 participants received placebo (13 female; age range, 11 years; mean, 24.38, SD = 3.22 years). The study was double blind. All participants were right-handed and had normal or corrected-to-normal visual acuity. None of the participants reported a history of neurological, psychiatric, or any other current medical problems. Two participants were excluded (one from the placebo and one from the citalopram groups) because of deterministic performance. Two further participants did not complete the task, one because of technical problems and the other because of gastrointestinal side effects after receiving citalopram.

### Experimental procedure for the drug study

Participants completed the task (see below) 60 min after receiving levodopa (150 mg + 37.5 mg benserazide; time to reach peak blood concentration after oral administration 1–2 h) or 180 min after receiving citalopram (24 mg in drops which is equivalent to 30 mg in tablet; time to reach peak blood concentration after oral administration 1–4 h). To ensure participants and investigators were blind to the treatment condition, each participant received one glass containing either citalopram or placebo. Two hours later, they received a second glass containing either placebo or levodopa and waited for another hour before engaging with the go/no-go learning task. The participants in the placebo group received a placebo in both occasions. Participants earned between £10 and £35, according to their performance in the current task. In addition, after performing the go/no-go task, participants engaged in an unrelated task and received between £5 and £20 for their participation *in* this second task. Participants completed a subjective state analogue-scales questionnaire on three occasions. We did not detect any difference in subjective ratings between treatment groups (data not shown).

### Behavioral paradigm

**Fig. 1:**
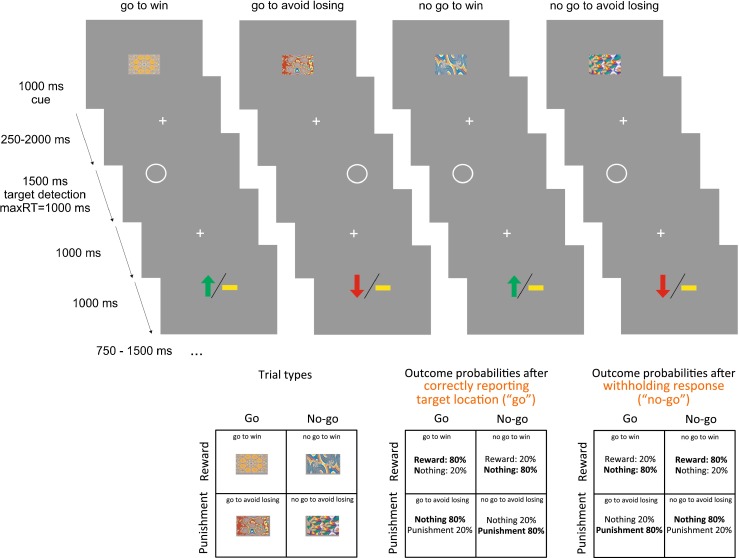
Experimental paradigm. On each trial, one of four possible fractal images indicated the combination between action (making a button press in go trials or withholding a button press in no-go trials) and valence at outcome (win or lose). Actions were required in response to a circle that followed the fractal image after a variable delay. On go trials, subjects indicated via a button press on which side of the screen the circle appeared. On no-go trials they withheld a response. After a brief delay, the outcome was presented: a *green upward arrow* indicated a win of £1 and a *red downward arrow* a loss of £1. A *horizontal bar* indicated of the absence of a win or a loss. On *go to win trials* a correct button press was rewarded, on *go to avoid losing trials* a correct button press avoided punishment, in *no-go to win trials* a correct withholding a button press led to reward, and in *no-go to avoid losing trials* a correct withholding a button press avoided punishment

We used the learning version of an experimental design that orthogonalizes action and valence (Guitart-Masip et al. 2012b). The trial timeline is displayed in Fig. 1. Each trial consisted of three events: a fractal cue, a target detection task, and a probabilistic outcome. At the beginning of each trial, one of four distinct fractal cues was presented which indicated whether the best choice in a subsequent target detection task was a go (emitting a button press to a target) or a no-go (withholding any response to a target). The fractal also reported the valence of any outcome consequent on the subject’s behavior (reward/no reward or punishment/no punishment). The meaning of fractal images (go to win; no-go to win; go to avoid losing; no-go to avoid losing) was randomized across participants. As in Guitart-Masip (2012b), but unlike Guitart-Masip et al. (2011), subjects had to learn these by trial and error. Participants were instructed that correct choice for each fractal image could be either go or no-go and about the probabilistic nature of the task.

The target was a circle on one side of the screen and was displayed for 1,500 ms starting 250 to 2,000 ms after the offset of the fractal image. Based on the fractal image, participants had to decide whether (go) or not (no-go) to press the key to indicate the target location. A response was classified as a correct go choice if participants pressed the key corresponding to the correct side within 1,000 ms after target onset, and a no-go choice otherwise. At 1,000 ms following offset of the target, the outcome was displayed for 1,000 ms: A green upward arrow indicated a £1 win; a red downwards arrow indicated a £1 loss, and a yellow horizontal bar indicated no win or loss. The outcome was probabilistic: In win trials, 80 % of correct choices and 20 % of incorrect choices were rewarded (the remaining 20 % of correct and 80 % of incorrect choices led to no outcome); in lose trials, 80 % of correct choices and 20 % of incorrect choices avoided punishment.

The task included 240 trials in total, i.e., 60 trials per condition. Before starting with the learning task, subjects performed 20 trials of the target detection task in order to get familiarized with the speed requirements.

### Behavioral data analysis

The behavioral data were analyzed using the statistics software SPSS, version 16.0. *The probability of correct choice* in the target detection task (correct button press for go conditions and correct omission of responses in no-go trials) were collapsed across time bins of ten trials per condition and were analyzed with a mixed ANOVA with time bins, action (go/no-go), and valence (win/lose) as within-subject factors and treatment (levodopa, citalopram, and placebo) as a between-subjects factor. Greenhouse-Geiser correction was applied when the sphericity assumption was violated.

### Reinforcement learning models

Following Guitart-Masip et al. (2012b), we built six nested models incorporating different instrumental and Pavlovian reinforcement-learning hypotheses and fit these to the observed behavioral data. All models assigned probabilities to each action *a*_t_ (here, go or no-go) on each trial *t*. These probabilities were based on action propensities *w*(*a*_t_,*s*_*t*_) that depended on the stimulus on that trial and which were passed through a squashed sigmoid function (Sutton and Barto 1998):

1 $$ p\left({a}_t|{s}_t\right)=\left[\frac{ \exp \left(\mathrm{w}\right.\left({a}_t,{s}_t\right)}{{\displaystyle {\sum}_{a\prime } \exp \left(\mathrm{w}\left(a\prime, {s}_t\right)\right)}}\right]\left(1-\xi \right)+\frac{\xi }{2} $$

The models differed in the construction of the action propensities and the value of the irreducible noise ξ. They allowed us to test the hypotheses that behavior was purely instrumental, or included a Pavlovian component (‘Pav’) which captures the critical coupling between affect and effect: that there were or were not asymmetries between the subjects’ sensitivities to reward versus punishment (rew/pun); that they had an intrinsic propensity to go versus no go (bias), or to repeat or avoid their previous choice (stick); and that there was or was not irreducible stochasticity (or trembling) in their behavior (noise).

More completely, ξ was kept at 0 for one of the models (RW) but was free to vary between 0 and 1 for all other models. For models RW and RW + noise, *w*(*a*,*s*) = *Q*(*a*,*s*) was based on a simple Rescorla-Wagner or delta rule update equation:

2 $$ {Q}_t\left({a}_t,{s}_t\right)={Q}_{t-1}\left({a}_t,{s}_t\right)+\varepsilon \left(\rho {r}_t-{Q}_{t-1}\left({a}_t,{s}_t\right)\right) $$

where *ε* is the learning rate. Reinforcements enter the equation through *r*_*t*_{−1,0,1} and *ρ* is a free parameter that determined the effective size of reinforcements. For some models (RW, RW + noise, and RW + noise + bias), there was only one value of *ρ* per subject. This meant that those models assumed that loss of a reward was equally as aversive as obtaining a punishment. Other models included different sensitivities to reward and punishment (RW(rew/pun) + noise + bias, RW(rew/pun) + noise + bias + Pav, and RW(rew/pun) + noise + bias + Pav + stick) allowing different values of the parameter *ρ* on reward-and-punishment trials, thus assuming that loss of a reward was *not* equally as aversive as obtaining a punishment.

Further models added extra factors to the action propensities. For models that contained a bias parameter, the action weight was modified to include a static bias parameter *b*:

3 $$ {w}_t\left(a,s\right)=\left\{\begin{array}{l}{Q}_t\left(a,s\right)+b\kern1em \mathrm{if}\kern0.5em a=\mathrm{go}\hfill \\ {}{Q}_t\left(a,s\right)\kern4em \mathrm{else}\hfill \end{array}\right. $$

For the model including a Pavlovian factor (RW(rew/pun) + noise + bias + Pav), the action weight consisted of three components:

4 $$ {w}_{\mathrm{t}}\left(a,s\right)=\left\{\begin{array}{l}{Q}_t\left(a,s\right)+b+\pi {V}_t(s)\kern1em \mathrm{if}\kern0.5em a=\mathrm{go}\hfill \\ {}{Q}_t\left(a,s\right)\kern8em \mathrm{else}\hfill \end{array}\right. $$

5 $$ {V}_t\left({s}_t\right)={V}_{t-1}\left({s}_t\right)+\varepsilon \left(\rho {r}_t-{V}_{t-1}\left({s}_t\right)\right) $$

where *π* was again a free parameter. Thus, for the “avoid loss” conditions, in which the *V*(*s*) would be non-positive, the Pavlovian parameter inhibited the go tendency in proportion to the negative value *V*(*s*) of the stimulus, while it similarly promoted the tendency to go in conditions in the “win” conditions.

For the model including stickiness (RW(rew/pun) + noise + bias + Pav + stick), the action weight consisted of four components:

6 $$ {w}_t\left(a,s\right)=\left\{\begin{array}{l}{Q}_t\left(a,s\right)+b+\pi {V}_t(s)+c{\chi}_{a=a\left(t-1\right)}\kern1em \mathrm{if}\kern0.5em a=\mathrm{go}\hfill \\ {}{Q}_t\left(a,s\right)+c{\chi}_{a=a\left(t-1\right)}\kern8em \mathrm{else}\hfill \end{array}\right. $$

where *c* is a free parameter that boosts or suppresses the action performed on the previous trial. This component was added because it is often found that subjects have a tendency either to repeat or avoid doing the same action twice (Lau and Glimcher 2005; Schoenberg et al. 2007; Rutledge et al. 2010) and dietary tryptophan depletion results in increased value independent of choice perseveration (Seymour et al. 2012).

As in previous reports (Guitart-Masip et al. 2012a; Huys et al. 2011), we used a hierarchical Type II Bayesian (or random effects) procedure using maximum likelihood to fit simple parameterized distributions for higher-level statistics of the parameters. Since the values of parameters for each subject are “hidden”, this employs the expectation–maximization procedure. On each iteration, the posterior distribution over the group for each parameter is used to specify the prior over the individual parameter fits on the next iteration. For each parameter, we used a single distribution for all participants. Therefore, the fitting procedure was blind to the existence of different treatment groups with putatively different parameter values. Before inference, all parameters except the action bias were suitably transformed to enforce constraints (log and inverse sigmoid transforms).

Models were compared using the *integrated* Bayesian Information Criterion (iBIC), where small iBIC values indicate a model that fits the data better after penalizing for the number of parameters. The iBIC is not the sum of individual likelihoods, but the integral of the likelihood function over the individual parameters (for details, see Huys et al. 2011). Comparing iBIC values is akin to a likelihood ratio test (Kass and Raftery 1995). The model fitting and selection procedures were verified on surrogate data generated from a known decision process (Electronic supplementary material figures 1 and 2).

The model parameters of the winning model were compared across treatment groups using a one-way ANOVA when these were normally distributed (the sensitivity to reward and the action bias) and the Kruskal–Wallis test when not normally distributed. Normality was assessed by means of Kolmogorov–Smirnov test. Independent sample *t* test or Mann–Whitney *U* test were used as post hoc test when appropriate.

## Results

### Levodopa and citalopram differentially impact on the effects of reward and punishment on go and no-go choices

A mixed ANOVA with time bins, action (go/no-go), and valence (win/lose) as within-subject factors, and treatment (levodopa, citalopram, and placebo) as a between-subjects factor revealed two key patterns across all participants as previously reported (Cavanagh et al. 2013; Guitart-Masip et al. 2012a, b). First, overall performance across the entire experiment was better in the go to win condition compared with the go to avoid losing condition and in the no-go to avoid losing condition when compared with no-go to win condition (see Table 1). This results in a significant action by valence interaction (F(1,85) = 69.29, *p* < 0.001), which is consistent with a Pavlovian process linking action to valence. Second, participants showed an overall better performance on go compared with no-go conditions reflected in a main effect of action (F(1,85) = 64.17, *p* < 0.001).

**Table 1:** Raw overall behavioral performance

	Go to win	Go to avoid	No-go to win	No-go to avoid
All groups	0.936 ± 0.011	0.819 ± 0.017	0.539 ± 0.04	0.794 ± 0.021
Placebo	0.955 ± 0.014	0.776 ± 0.04	0.536 ± 0.073	0.856 ± 0.03
Levodopa	0.917 ± 0.027	0.837 ± 0.063	0.648 ± 0.063	0.773 ± 0.038
Citalopram	0.936 ± 0.017	0.843 ± 0.014	0.428 ± 0.07	0.754 ± 0.041

Mean (±SEM) proportion of successful trials across the whole sample and for each treatment group separately. A successful trial involved a correct response within the response deadline for the go trials (go-to-win and go-to-avoid-losing) and withholding response on the no-go trials (no-go-to-win and no-go-to-avoid-losing). For go trials, anticipation of punishment decreased the proportion of successful trials whereas for no-go trials anticipation of reward decreased the proportion of successful trials. Note that for each experimental condition, we only provide the overall probability of a correct response across the entire experiment (collapsing across time bins) because we did not detect any time bin × drug interaction

**Fig. 2:**
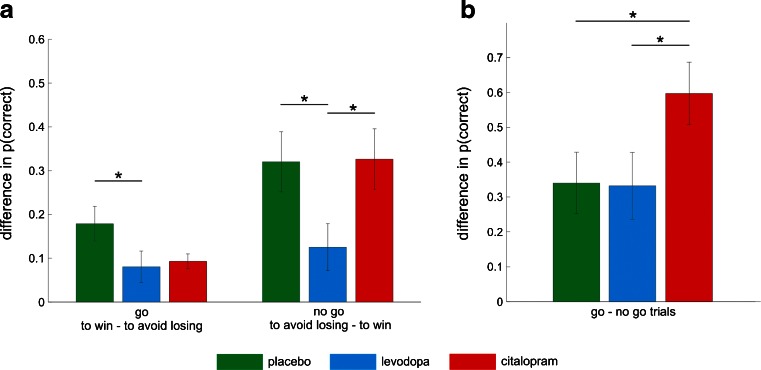
Effects of levodopa and citalopram on choice performance. **a** Mean (±SEM) difference in proportion of correct trials between go to win and go to avoid losing (*left*) and between no-go to avoid losing and no-go to win (*right*). These two different scores represent the two terms of the interaction between action and valence in choice accuracy. *Green* represents the differential scores for the placebo group, blue for the levodopa group, and *red* for the citalopram group. Levodopa decreased the disadvantage of learning go to avoid losing when compared with go to win observed in the placebo group. Levodopa also decreased the disadvantage of learning no-go to win when compared with no-go to avoid losing observed both in the placebo and the citalopram groups. Post hoc comparisons were implemented by means of *t* test: *p < 0.05. **b** Mean (±SEM) difference in proportion of correct trials between go and no-go conditions, that is, the main effect of action. *Green* represents the differential scores for the placebo group, *blue* for the levodopa group, and *red* for the citalopram group. Citalopram increased the advantage of learning the go when compared with the no-go conditions observed in the levodopa and the placebo groups. Post hoc comparisons were implemented by means of *t* test: *p < 0.05

These effects were modulated by the pharmacological treatments. First, there was a significant action by valence by treatment interaction (F(2,85) = 3.82, *p* = 0.026) which was driven by the levodopa group. Levodopa decreased the difference in overall performance between the go to win and the go to avoid losing conditions and between the no-go to avoid losing and the no-go to win conditions (see Fig. 2a). Second, we also observed a trend for a treatment by action effect (F(2,85) = 2.7, *p* = 0.073) driven by an enhanced main effect of action in the citalopram group (see Fig. 2b). Interestingly, the two drug treatments only differed significantly in the no-go to win condition in which participants who received levodopa showed higher performance than participants who received citalopram (*t*(57) = 2.34; *p* = 0.023). This is a key result as it allows us to distinguish between a decoupling of action and valence from a facilitation of go responses regardless of valence. Whereas a decoupling of action and valence is associated with simultaneous facilitation of the go to avoid losing and the no-go to win conditions, a facilitation of go responses regardless of valence is associated with facilitation of the go to avoid losing condition but impairment of the no-go to win condition.

Finally, we found a main effect of time (F(2.9,242.3) = 199.1, *p* < 0.001) in the absence of any interaction between treatment × time (*p* > 0.05). The learning curves for each trial type because in each treatment group are available in the supplementary material. These drug effects are unlikely to be related to unspecific arousal effects because we did not find any difference in subjective ratings between treatment groups.

### Effects of drugs on model parameters

We examined these effects in more detail using reinforcement-learning models to parameterize a fine-grained account of the interaction between action and valence while participants learnt the reward structure of the environment. We built a nested collection of models incorporating different instrumental and Pavlovian reinforcement learning hypotheses which have been discussed in detail previously (Guitart-Masip et al. 2012b). In brief, the base model (RW) is purely instrumental, learning action values independently of outcome valence using the Rescorla-Wagner rule. This model was augmented in successive steps: In RW + noise, the model includes irreducible choice noise to the instrumental system; in RW + noise + bias, the model further includes a value-independent action bias that promotes or suppresses go choices equally in all conditions; in RW(rew/pun) + noise + bias, the instrumental system includes separate reward and punishment sensitivities which implies that losing a reward was *not* equally as aversive as getting a punishment; in RW(rew/pun) + noise + bias + Pav, the model further includes a (Pavlovian) parameter the adds a fraction of the state value into the action values learned by the instrumental system, thus effectively coupling action and valence during learning; finally, the last model RW(rew/pun) + noise + bias + Pav + stick includes a value-independent perseveration parameter that boosts or suppresses the action performed on the previous trial.

The most parsimonious model turned out to be RW(rew/pun) + noise + bias + Pav. Critically, this includes a Pavlovian bias parameter that increased the probability of go choices proportionally to the overall (action-independent) state value of each stimulus. This Pavlovian bias parameter thus increased the probability of go choices when the state values were positive (winning conditions) and decreased it when the state values were negative (avoid losing conditions). The fact that the winning model included a Pavlovian component demonstrates that the observed learning behavior is best characterized when including a component that couples action and valence. Previous incarnations of the learning version of the task have also implicated this model (Cavanagh et al. 2013; Guitart-Masip et al. 2012b), except that including separate reward and punishment sensitivity parameters improved the model fit independently from the Pavlovian parameter (see Table 2). We did not consider this possibility in our previous report where the winning model only included one single reinforcement sensitivity parameter (Guitart-Masip et al. 2012b).

**Table 2:** Model comparison

	Number of parameters	iBIC
RW	2	17,120
RW + noise	3	16,998
RW + noise + bias	4	15,398
RW(rew/pun) + noise + bias	5	14,765
RW(rew/pun) + noise + bias + Pav	6	14,011
RW(rew/pun) + noise + bias + Pav + Stick	7	14,016

**Fig. 3:**
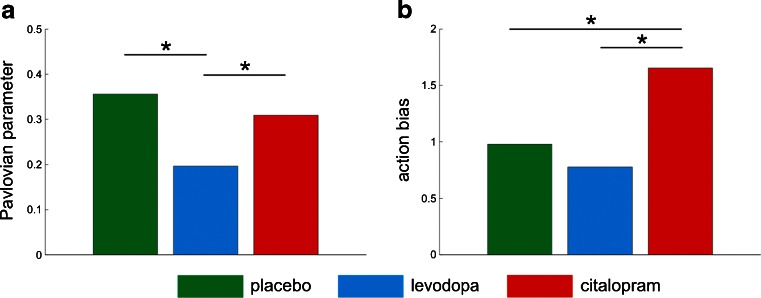
Effects of levodopa and citalopram on model parameters. **a** Maximum a posteriori (MAP) median parameter estimates of the best model for the Pavlovian parameter. *Green* represents the differential scores for the placebo group, *blue* for the levodopa group, and *red* for the citalopram group. Levodopa decreased the Pavlovian parameter when compared with placebo and citalopram. Post hoc comparisons were implemented by means of Mann–Whitney *U* test: *p < 0.05. **b** Maximum a posteriori (MAP) median parameter estimates of the best model for the action bias parameter. *Green* represents the differential scores for the placebo group, *blue* for the levodopa group, and *red* for the citalopram group. Citalopram increased the action bias parameter when compared with placebo and levodopa. Post hoc comparisons were implemented by means of *t* test: *p < 0.05

Once we identified that the model that best characterized the observed learning asymmetries includes an instrumental learning system with irreducible noise and a value-independent action bias along with a Pavlovian system that effectively couples action and valence during learning, we examined whether the pharmacological manipulations had any effect on the parameters of the model. For each parameter of the winning model, the median and 25th and 75th posterior percentiles across the whole sample are displayed in Table 3. We detected a difference between treatment groups on the Pavlovian parameter (Kruskal Wallis test *χ* ^2^(2) = 6.5, *p* = 0.039) and the bias parameter (one way ANOVA F(2,85) = 3.94; *p* = 0.023). As shown in Fig. 3a, levodopa decreased the Pavlovian parameter compared with placebo (Mann–Whitney *U* test Z = 2.14, *p* = 0.033) and to citalopram (Mann–Whitney *U* test Z = 2.2, *p* = 0.028). On the other hand, citalopram increased the action bias parameter compared with placebo (Fig. 3b, *t* test t(56) = 2.62, *p* = 0.011) and to levodopa (*t* test t(57) = 2.27, *p* = 0.027). The effects of citalopram were not related to changes in stickiness.

**Table 3:** Parameters of the winning model

	Percentile 25	Median	Percentile 75
Sensitivity to reward	6.07	12.59	18.99
Sensitivity to punishment	6.2	9.33	12.86
Learning rate	0.08	0.18	0.31
Noise	0.93	0.96	0.98
Pavlovian	0.16	0.27	0.48
Action bias	0.35	1.14	2.21

A recent study showed that dietary tryptophan depletion increased a value-independent choice perseveration or stickiness (Seymour et al. 2012). To rule out the possibility that the effect of citalopram is explained by increased stickiness, we compared the action bias and the stickiness posterior parameter estimates for the model including the stickiness parameter (despite the fact that this model did not provide a better account of the data). We did not find any significant difference in the stickiness parameter between the placebo and the citalopram group (Mann–Whitney *U* test *Z* = 0.63, *p* = 0.53), whereas the difference in action bias parameter remained significant (*t*(56) = 2.32; *p* = 0.024).

## Discussion

The current data reveal differential, but not opponent, effects of levodopa (l-DOPA) and citalopram, on instrumental control, which we assume arise from effects on dopamine and serotonin, respectively. As in previous experiments, we detected a striking asymmetry during instrumental learning in the placebo group, whereby participants learnt better to emit a behavioral response in anticipation of reward and learnt better to withhold a response in anticipation of punishment. This asymmetry was attenuated post-administration of levodopa, and our computational analysis indicated this was mediated by a decreased influence of a Pavlovian controller that corrupts instrumental control. Conversely, administration of citalopram increased the propensity to perform go choices regardless of outcome valence, as reflected in an increased magnitude of the value independent action bias parameter.

A wealth of studies suggests at least three different roles dopamine might play in guiding behavior. Two of these roles relate to value/action learning via reward prediction errors (Bayer and Glimcher 2005; Montague et al. 1996; Morris et al. 2006; Schultz et al. 1997) and action invigoration via phasic (Satoh et al. 2003) and tonic release (Salamone et al. 1994) in Pavlovian (Lex and Hauber 2008; Parkinson et al. 2002) and instrumental (Dayan 2012; Guitart-Masip et al. 2012a; Niv et al. 2007) contexts. Both are tied to dopamine’s effects in ventral and dorsal striatum, for example, through influencing the balance between go-related direct and no-go-related indirect pathways (Frank et al. 2004; Wickens et al. 2007).

The role of dopamine in learning provides a plausible mechanism for acquisition of active responses through positive reinforcement and passive responses through punishment. According to a prevalent view in reinforcement learning and decision making, dopamine neurons signal reward prediction error signals (Bayer and Glimcher 2005; Montague et al. 1996; Schultz et al. 1997) in the form of phasic bursts for positive prediction errors and dips below baseline for negative prediction errors (Bayer et al. 2007), to target structures including the striatum (McClure et al. 2003; O’Doherty et al. 2003, 2004; Pessiglione et al. 2006). In the striatum, increases of dopamine when an unexpected reward is obtained reinforce the direct pathway and generate go choices, while dips in dopamine levels when an unexpected punishment is obtained reinforce the indirect pathway and generate no-go choices (Frank et al. 2007; Frank et al. 2004; Hikida et al. 2010; Wickens et al. 2007). However, this framework provides no clear mechanism for learning to go to avoid losing or no-go to win. For this reason, we have argued that a coupling between action and valence within the corticostriatal system could underlie the strong Pavlovian influences in instrumental learning observed in our task (Guitart-Masip et al. 2012b). However, if l-DOPA through its impact on dopamine mediated this function, then we would expect increased asymmetries in task performance, rather than the decrease in asymmetric learning that we observed.

It is known that dopamine depletion results in decreased motor activity and decreased motivated behavior (Palmiter 2008; Ungerstedt 1971), along with decreased vigor or motivation to work for rewards in demanding reinforcement schedules (Niv et al. 2007; Salamone et al. 2005). Conversely, boosting dopamine levels with levodopa invigorates motor responding in healthy humans (Guitart-Masip et al. 2012a), possibly by increasing the invigorating effects exercised by average reward rate on response time (Beierholm et al. 2013). Additionally, enhancing dopamine in the nucleus accumbens increases vigor in appetitive Pavlovian-instrumental transfer (Lex and Hauber 2008; Parkinson et al. 2002) and invigorates appetitive instrumental actions (Taylor and Robbins 1984, 1986). However, if this was the predominant effect of levodopa, then we would have expected increased action biases and/or Pavlovian influences, which in fact did not arise.

A third potential role for dopamine arises from its influence on the balance between different sorts of control (Hitchcott et al. 2007). This function can be achieved, for instance, by facilitating the operation of prefrontal processes such as working memory or rule learning (Clatworthy et al. 2009; Cools and D’Esposito 2011; Mehta et al. 2005; Williams and Goldman-Rakic 1995), perhaps reducing the error in their outputs and thereby increasing their influence on behavior (Daw et al. 2005). An alternative mechanism by which dopamine may arbitrate between different sorts of control is through recruitment of the prefrontal–subthalamic nucleus pathway (Aron and Poldrack 2006; Fleming et al. 2010) to raise a decision threshold within the basal ganglia and thereby prevent execution of a biased decision computed in the striatum (Cavanagh et al. 2011; Frank 2006; Zaghloul et al. 2012).

The involvement of a prefrontal mechanism in overcoming a Pavlovian interference is supported by recent evidence that theta power over midline frontal sensors exert a moderating effect on Pavlovian influences on trials where Pavlovian and instrumental control conflict (Cavanagh et al. 2013). Furthermore, successfully learning to perform the no-go conditions in our task is known to involve a recruitment of inferior frontal gyrus (Guitart-Masip et al. 2012b). A related finding is the observation that levodopa increases the degree to which young healthy participants employ model-based, as opposed to model-free, control in a two-step choice task (Wunderlich et al. 2012), a task sensitive to manipulations of working memory load (Otto et al. 2013). Concomitantly, depleting dopamine can boost model-free control (de Wit et al. 2012). Thus, the effects we observe may in part depend on dopamine’s actions on functions implemented in prefrontal cortex (Hitchcott et al. 2007). However, future imaging experiments are required to localize the anatomical site and component processes that account for the observed effects of levodopa seen in the context of the current task.

Two previous experiments from our laboratory also suggest a role for dopamine in decoupling the instrumental and Pavlovian learning systems in the current task, although they do not pin down the site of its action. In one, older adults showed the same asymmetric association between action and valence that we report in younger adults. Furthermore, in older adults, we also found that the integrity of the substantia nigra/ventral tegmental area, as measured with structural magnetic resonance imaging, is positively correlated with performance in the no-go to win condition, the condition with highest Pavlovian and instrumental conflict and worst performance (Chowdhury et al. 2013). In the other, we studied the effects of levodopa on striatal BOLD signal in subjects who had explicitly been taught the contingencies of the task rather than having to learn them for themselves. In this context, striatal BOLD responses are dominated by action requirements (go > no-go) rather than valence (Guitart-Masip et al. 2011). However, after administering levodopa, there was decreased BOLD response in the no-go to win condition along with increased BOLD in the go to win case (Guitart-Masip et al. 2012a). This neuronal effect may be a homologue of the decrease in a Pavlovian bias observed in the current study after administration of levodopa and suggest yet another mechanism by which a supposedly prefrontal effect of levodopa may decrease the Pavlovian bias, namely by modulation of model-free representations of prediction errors at a subcortical level (Daw et al. 2011; Doll et al. 2011).

There is good evidence of a role for serotonin in inhibition (Soubrie 1986) with serotonin depletion in rats impairing their ability to withhold action in a symmetrically rewarded go/no-go task (Harrison et al. 1999) and increasing the number of premature responses in the five choice reaction time task (Carli and Samanin 2000; Harrison et al. 1997). Furthermore, selectively inhibiting serotonin neurons and preventing serotonin increases in the prefrontal cortex abolishes the ability of rats to wait for rewards during a long delay (Miyazaki et al. 2012). Nevertheless, involvement of serotonin in behavioral inhibition is typically complicated (Cools et al. 2011; Drueke et al. 2010). A previous study found that dietary tryptophan depletion abolishes punishment-induced inhibition (as measured with reaction times) akin to the disadvantage of performing a go response in the avoid losing condition when compared with the winning condition (Crockett et al. 2009), and recent follow up study suggests that this effect is driven by a Pavlovian mechanism (Crockett et al. 2012).

By themselves, these results depict a complex picture without any clear expectation about the effects of citalopram. Citalopram is a selective serotonin reuptake inhibitor, whose direct effect is locally increased serotonin availability. However, acute citalopram administration results in decreased total postsynaptic serotonin availability, at least at the cortical level (Selvaraj et al. 2012), possibly through a presynaptic inhibitory mechanism (Artigas et al. 1996; Hajos et al. 1995). Citalopram is likely to have weaker effects than dietary tryptophan depletion, and the study suggesting a Pavlovian source for punishment-induced inhibition (Crockett et al. 2012) is most equivalent to our steady state or instructed study, where we did not observe an effect of citalopram (Guitart-Masip et al. 2012a).

Our data suggest that the effects of citalopram were confined to a behavioral inhibition independent of valence, with no apparent modulation of the strength of the Pavlovian parameter. Given the lack of effect on learning, one might have thought that citalopram, as in (Guitart-Masip et al. 2012a), would have had no effect at all. However, two functional differences between taught and learnt versions of our task are worth noting. First, subjects in the learning version have to overcome a value-independent action bias that may have arisen because participants performed 20 target detection trials in order to get familiarized with the speed requirements, before embarking on the learning task; second, as a result of this, they choose go more frequently, rendering themselves more susceptible to the effects of perseveration. Our computational model clearly shows that the effects of citalopram are captured by an increase in action bias, which may explain why we only find an effect of citalopram when the task involves the requirement to overcome an action bias.

The current data again highlight the importance of orthogonally manipulating action requirements and outcome valence if one wants to reveal the full complexity of the roles played by dopamine and serotonin in instrumental learning. We found that boosting dopamine via levodopa decreases the pervasive coupling between Pavlovian and instrumental control systems. On the other hand, our data reveal the differential, but not opponent, effect of reducing motor inhibition by manipulating serotonin via citalopram. Overall, the data speak to a need for wider panoply of methods for manipulating dopamine and serotonin in human subjects, allowing the more fine-grained range of effects evident in more pharmacologically and spatially restricted studies in animals to be examined.

## Electronic supplementary material

Below is the link to the electronic supplementary material.

ESM 1 (DOCX 548 kb)

## References

[CR1] Aron AR, Poldrack RA (2006). Cortical and subcortical contributions to Stop signal response inhibition: role of the subthalamic nucleus. J Neurosci Off J Soc Neurosci.

[CR2] Artigas F, Romero L, de Montigny C, Blier P (1996). Acceleration of the effect of selected antidepressant drugs in major depression by 5-HT1A antagonists. Trends Neurosci.

[CR3] Bayer HM, Glimcher PW (2005). Midbrain dopamine neurons encode a quantitative reward prediction error signal. Neuron.

[CR4] Bayer HM, Lau B, Glimcher PW (2007) Statistics of midbrain dopamine neuron spike trains in the awake primate. J Neurophys 98:1428–1439 10.1152/jn.01140.2006 17615124

[CR5] Beierholm U, Guitart-Masip M, Economides M, Chowdhury R, Duzel E, Dolan R, Dayan P (2013) Dopamine modulates reward-related vigor. Neuropsychopharmacology 38:1495–1503 10.1038/npp.2013.48 PMC3682144 23419875

[CR6] Berridge KC, Robinson TE (1998). What is the role of dopamine in reward: hedonic impact, reward learning, or incentive salience?. Brain Res Brain Res Rev.

[CR7] Boureau YL, Dayan P (2011). Opponency revisited: competition and cooperation between dopamine and serotonin. Neuropsychopharmacol Off Publ Am Coll Neuropsychopharmacol.

[CR8] Breland K, Breland M (1961). The misbehavior of organisms. Am Psychol.

[CR9] Carli M, Samanin R (2000). The 5-HT(1A) receptor agonist 8-OH-DPAT reduces rats’ accuracy of attentional performance and enhances impulsive responding in a five-choice serial reaction time task: role of presynaptic 5-HT(1A) receptors. Psychopharmacology.

[CR10] Cavanagh JF, Eisenberg E, Guitart-Masip M, Huys Q, Frank MJ (2013) Frontal Theta Overrides Pavlovian Learning Biases. J Neurosci Off J Soc Neurosci 33:8541-8548 10.1523/JNEUROSCI.5754-12.2013 PMC3707146 23658191

[CR11] Cavanagh JF, Wiecki TV, Cohen MX, Figueroa CM, Samanta J, Sherman SJ, Frank MJ (2011). Subthalamic nucleus stimulation reverses mediofrontal influence over decision threshold. Nat Neurosci.

[CR12] Chowdhury R, Guitart-Masip M, Lambert C, Dolan R, Duzel E (2013) Structural integrity of the substantia nigra and subthalamic nucleus determines the flexibility of instrumental learning in old age. Neurobiol Aging 34:2261-2270 10.1016/j.neurobiolaging.2013.03.030 PMC3713434 23623600

[CR13] Clatworthy PL, Lewis SJ, Brichard L, Hong YT, Izquierdo D, Clark L, Cools R, Aigbirhio FI, Baron JC, Fryer TD, Robbins TW (2009). Dopamine release in dissociable striatal subregions predicts the different effects of oral methylphenidate on reversal learning and spatial working memory. J Neurosci Off J Soc Neurosci.

[CR14] Cools R, D’Esposito M (2011). Inverted-U-shaped dopamine actions on human working memory and cognitive control. Biol Psychiatr.

[CR15] Cools R, Nakamura K, Daw ND (2011). Serotonin and dopamine: unifying affective, activational, and decision functions. Neuropsychopharmacol Off Publ Am Coll Neuropsychopharmacol.

[CR16] Crockett MJ, Clark L, Apergis-Schoute AM, Morein-Zamir S, Robbins TW (2012). Serotonin modulates the effects of Pavlovian aversive predictions on response vigor. Neuropsychopharmacol Off Publ Am Coll Neuropsychopharmacol.

[CR17] Crockett MJ, Clark L, Robbins TW (2009). Reconciling the role of serotonin in behavioral inhibition and aversion: acute tryptophan depletion abolishes punishment-induced inhibition in humans. J Neurosci Off J Soc Neurosci.

[CR18] Daw ND, Doya K (2006). The computational neurobiology of learning and reward. Curr Opin in Neurobiol.

[CR19] Daw ND, Gershman SJ, Seymour B, Dayan P, Dolan RJ (2011). Model-based influences on humans’ choices and striatal prediction errors. Neuron.

[CR20] Daw ND, Niv Y, Dayan P (2005). Uncertainty-based competition between prefrontal and dorsolateral striatal systems for behavioral control. Nat Neurosci.

[CR21] Dayan P (2012). Twenty-five lessons from computational neuromodulation. Neuron.

[CR22] Dayan P, Huys QJ (2009). Serotonin in affective control. Annu Rev Neurosci.

[CR23] Dayan P, Niv Y, Seymour B, Daw ND (2006). The misbehavior of value and the discipline of the will. Neural Netw.

[CR24] de Wit S, Standing HR, Devito EE, Robinson OJ, Ridderinkhof KR, Robbins TW, Sahakian BJ (2012). Reliance on habits at the expense of goal-directed control following dopamine precursor depletion. Psychopharmacology.

[CR25] Dickinson A, Balleine B (2002). The role of learning in motivation.

[CR26] Doll BB, Hutchison KE, Frank MJ (2011). Dopaminergic genes predict individual differences in susceptibility to confirmation bias. J Neurosci Off J Soc Neurosci.

[CR27] Drueke B, Boecker M, Schlaegel S, Moeller O, Hiemke C, Grunder G, Gauggel S (2010). Serotonergic modulation of response inhibition and re-engagement? Results of a study in healthy human volunteers. Hum Psychopharmacol.

[CR28] Flagel SB, Clark JJ, Robinson TE, Mayo L, Czuj A, Willuhn I, Akers CA, Clinton SM, Phillips PE, Akil H (2011). A selective role for dopamine in stimulus-reward learning. Nature.

[CR29] Fleming SM, Thomas CL, Dolan RJ (2010). Overcoming status quo bias in the human brain. Proc Natl Acad Sci U S A.

[CR30] Frank MJ (2006). Hold your horses: a dynamic computational role for the subthalamic nucleus in decision making. Neural Netw.

[CR31] Frank MJ, Seeberger LC, O’Reilly RC (2004). By carrot or by stick: cognitive reinforcement learning in parkinsonism. Science.

[CR32] Frank MJ, Moustafa AA, Haughey HM, Curran T, Hutchison KE (2007) Genetic triple dissociation reveals multiple roles for dopamine in reinforcement learning. Proc Natl Acad Sci U S A 104:16311–16316 10.1073/pnas.0706111104 PMC2042203 17913879

[CR33] Gray JA, McNaughton M (2000). The neuropsychology of anxiety: an inquiry into the function of the septohippocampal system.

[CR34] Guitart-Masip M, Fuentemilla L, Bach DR, Huys QJ, Dayan P, Dolan RJ, Duzel E (2011). Action dominates valence in anticipatory representations in the human striatum and dopaminergic midbrain. J Neurosci Off J Soc Neurosci.

[CR35] Guitart-Masip M, Chowdhury R, Sharot T, Dayan P, Duzel E, Dolan RJ (2012). Action controls dopaminergic enhancement of reward representations. Proc Natl Acad Sci U S A.

[CR36] Guitart-Masip M, Huys QJ, Fuentemilla L, Dayan P, Duzel E, Dolan RJ (2012). Go and no-go learning in reward and punishment: interactions between affect and effect. NeuroImage.

[CR37] Hajos M, Gartside SE, Sharp T (1995). Inhibition of median and dorsal raphe neurones following administration of the selective serotonin reuptake inhibitor paroxetine. Naunyn Schmiedeberg’s Arch Pharmacol.

[CR38] Harrison AA, Everitt BJ, Robbins TW (1997). Doubly dissociable effects of median- and dorsal-raphe lesions on the performance of the five-choice serial reaction time test of attention in rats. Behav Brain Res.

[CR39] Harrison AA, Everitt BJ, Robbins TW (1999). Central serotonin depletion impairs both the acquisition and performance of a symmetrically reinforced go/no-go conditional visual discrimination. Behav Brain Res.

[CR40] Hikida T, Kimura K, Wada N, Funabiki K, Nakanishi S (2010) Distinct roles of synaptic transmission in direct and indirect striatal pathways to reward and aversive behavior. Neuron 66:896–907 10.1016/j.neuron.2010.05.011 20620875

[CR41] Hitchcott PK, Quinn JJ, Taylor JR (2007). Bidirectional modulation of goal-directed actions by prefrontal cortical dopamine. Cereb Cortex.

[CR42] Huys Q, Cools R, Golzer M, Friedel E, Heinz A, Dolan R, Dayan P (2011) Disentangling the roles of approach, activation and valence in instrumental and pavlovian responding. PLoS Comput Biol 7:e1002028 10.1371/journal.pcbi.1002028 PMC3080848 21556131

[CR43] Kass R, Raftery A (1995) Bayes factors. Journal of the American Statistical Association 106:1291-1303

[CR44] Koller WC, Rueda MG (1998) Mechanism of action of dopaminergic agents in Parkinson’s disease. Neurology 50: S11-4; discussion S44-8 10.1212/wnl.50.6_suppl_6.s11 9633680

[CR45] Lau B, Glimcher PW (2005) Dynamic response-by-response models of matching behavior in rhesus monkeys. J Exp Anal Behav 84:555–579 10.1901/jeab.2005.110-04 PMC1389781 16596980

[CR46] Lex A, Hauber W (2008). Dopamine D1 and D2 receptors in the nucleus accumbens core and shell mediate Pavlovian-instrumental transfer. Learn Mem.

[CR47] Mehta MA, Gumaste D, Montgomery AJ, McTavish SF, Grasby PM (2005). The effects of acute tyrosine and phenylalanine depletion on spatial working memory and planning in healthy volunteers are predicted by changes in striatal dopamine levels. Psychopharmacology.

[CR48] McClure SM, Berns GS, Montague PR (2003) Temporal prediction errors in a passive learning task activate human striatum. Neuron 38:339–46 10.1016/s0896-6273(03)00154-5 12718866

[CR49] Miyazaki KW, Miyazaki K, Doya K (2012). Activation of dorsal raphe serotonin neurons is necessary for waiting for delayed rewards. J Neurosci Off J Soc Neurosci.

[CR50] Montague PR, Dayan P, Sejnowski TJ (1996). A framework for mesencephalic dopamine systems based on predictive Hebbian learning. J Neurosci Off J Soc Neurosci.

[CR51] Morris G, Nevet A, Arkadir D, Vaadia E, Bergman H (2006). Midbrain dopamine neurons encode decisions for future action. Nat Neurosci.

[CR52] Niv Y, Daw ND, Joel D, Dayan P (2007). Tonic dopamine: opportunity costs and the control of response vigor. Psychopharmacology.

[CR53] O’Doherty JP, Dayan P, Friston K, Critchley H, Dolan RJ (2003) Temporal difference models and reward-related learning in the human brain. Neuron 38:329–37 10.1016/s0896-6273(03)00169-7 12718865

[CR54] O’Doherty J, Dayan P, Schultz J, Deichmann R, Friston K, Dolan RJ (2004) Dissociable roles of ventral and dorsal striatum in instrumental conditioning. Science 304:452–454 10.1126/science.1094285 15087550

[CR55] Otto RA, Gershman SJ, Markman AB, Daw N (2013) The curse of planning: dissecting multiple reinforcement learning systems by taxing the central executive. Psychol Sci 24:751–761 10.1177/0956797612463080 PMC3843765 23558545

[CR56] Palmiter RD (2008). Dopamine signaling in the dorsal striatum is essential for motivated behaviors: lessons from dopamine-deficient mice. Ann N Y Acad Sci.

[CR57] Parkinson JA, Dalley JW, Cardinal RN, Bamford A, Fehnert B, Lachenal G, Rudarakanchana N, Halkerston KM, Robbins TW, Everitt BJ (2002). Nucleus accumbens dopamine depletion impairs both acquisition and performance of appetitive Pavlovian approach behaviour: implications for mesoaccumbens dopamine function. Behav Brain Res.

[CR58] Parkinson JA, Olmstead MC, Burns LH, Robbins TW, Everitt BJ (1999). Dissociation in effects of lesions of the nucleus accumbens core and shell on appetitive pavlovian approach behavior and the potentiation of conditioned reinforcement and locomotor activity by d-amphetamine. J Neurosci Off J Soc Neurosci.

[CR59] Pessiglione M, Seymour B, Flandin G, Dolan RJ, Frith CD (2006) Dopamine-dependent prediction errors underpin reward-seeking behaviour in humans. Nature 442:1042–1045 10.1038/nature05051 PMC2636869 16929307

[CR60] Rutledge RB, Lazzaro SC, Lau B, Myers CE, Gluck MA, Glimcher PW (2009) Dopaminergic drugs modulate learning rates and perseveration in Parkinson’s patients in a dynamic foraging task. J Neurosci 29:15104–15114 10.1523/JNEUROSCI.3524-09.2009 PMC3376711 19955362

[CR61] Salamone JD, Correa M, Farrar A, Mingote SM (2007). Effort-related functions of nucleus accumbens dopamine and associated forebrain circuits. Psychopharmacology.

[CR62] Salamone JD, Correa M, Mingote SM, Weber SM (2005). Beyond the reward hypothesis: alternative functions of nucleus accumbens dopamine. Curr Opin Pharmacol.

[CR63] Salamone JD, Cousins MS, McCullough LD, Carriero DL, Berkowitz RJ (1994). Nucleus accumbens dopamine release increases during instrumental lever pressing for food but not free food consumption. Pharmacol Biochem Behav.

[CR64] Satoh T, Nakai S, Sato T, Kimura M (2003). Correlated coding of motivation and outcome of decision by dopamine neurons. J Neurosci Off J Soc Neurosci.

[CR65] Schonberg T, Daw ND, Joel D, O’Doherty JP (2007) Reinforcement learning signals in the human striatum distinguish learners from nonlearners during reward-based decision making. J Neurosci 27:12860–12867 10.1523/JNEUROSCI.2496-07.2007 PMC6673291 18032658

[CR66] Schultz W, Dayan P, Montague PR (1997). A neural substrate of prediction and reward. Science.

[CR67] Selvaraj S, Faulkner P, Mouchlianitis E, Roiser JP, Howes O (2012). Effects of citalopram on serotonin neurotransmission. Mol Psychiatry.

[CR68] Seymour B, Daw ND, Roiser JP, Dayan P, Dolan R (2012). Serotonin selectively modulates reward value in human decision-making. J Neurosci Off J Soc Neurosci.

[CR69] Soubrie P (1986). Reconciling the role of central serotonin neurons in human and animal behavior. Behav Brain Sci.

[CR70] Spinks D, Spinks G (2002). Serotonin reuptake inhibition: an update on current research strategies. Curr Med Chem.

[CR71] Sutton RS, Barto AG (1998). Reinforcement learning: an introduction.

[CR72] Taylor JR, Robbins TW (1984). Enhanced behavioural control by conditioned reinforcers following microinjections of d-amphetamine into the nucleus accumbens. Psychopharmacology.

[CR73] Taylor JR, Robbins TW (1986). 6-Hydroxydopamine lesions of the nucleus accumbens, but not of the caudate nucleus, attenuate enhanced responding with reward-related stimuli produced by intra-accumbens d-amphetamine. Psychopharmacology.

[CR74] Ungerstedt U (1971). Adipsia and aphagia after 6-hydroxydopamine induced degeneration of the nigro-striatal dopamine system. Acta Physiol Scand Suppl.

[CR75] Wickens JR, Budd CS, Hyland BI, Arbuthnott GW (2007). Striatal contributions to reward and decision making: making sense of regional variations in a reiterated processing matrix. Ann N Y Acad Sci.

[CR76] Williams GV, Goldman-Rakic PS (1995). Modulation of memory fields by dopamine D1 receptors in prefrontal cortex. Nature.

[CR77] Wunderlich K, Smittenaar P, Dolan RJ (2012). Dopamine enhances model-based over model-free choice behavior. Neuron.

[CR78] Zaghloul KA, Weidemann CT, Lega BC, Jaggi JL, Baltuch GH, Kahana MJ (2012). Neuronal activity in the human subthalamic nucleus encodes decision conflict during action selection. J Neurosci Off J Soc Neurosci.

