# Supplementary material for: Differential, but not opponent, effects of l-DOPA and citalopram on action learning with reward and punishment
Source: Psychopharmacology (Berl). 2013 Nov 15;231(5):955–66. doi: 10.1007/s00213-013-3313-4 (PMC3923110; doi:10.1007/s00213-013-3313-4)
Supplement: Supplementary file 1 — (DOCX 548 kb) [file 213_2013_3313_MOESM1_ESM.docx]

**SUPPLEMENTARY MATERIAL**

**Supplemental figure 1: testing the model fitting with surrogated data**

We generated 100 data sets per participant using the parameters of the winning model (RW(rew/pun) +noise+bias+Pav). The mean of the resulting surrogate data is shown in red in the attached figure (A-D) and superimposed on the real data of all participants (go responses in white) and the mean average probability of a go response in black. We then fitted each of the 100 datasets using the same EM algorithm that was used to fit the real data. In panel E we show all the inferred parameters plotted against the real parameter used to generate the surrogate data. In panel F we show the mean correlation coefficients across the samples for each parameter (sensitivity to reward, sensitivity to punishment, learning rate, Pavlovian parameter, irreducible noise, action bias) separately.

**
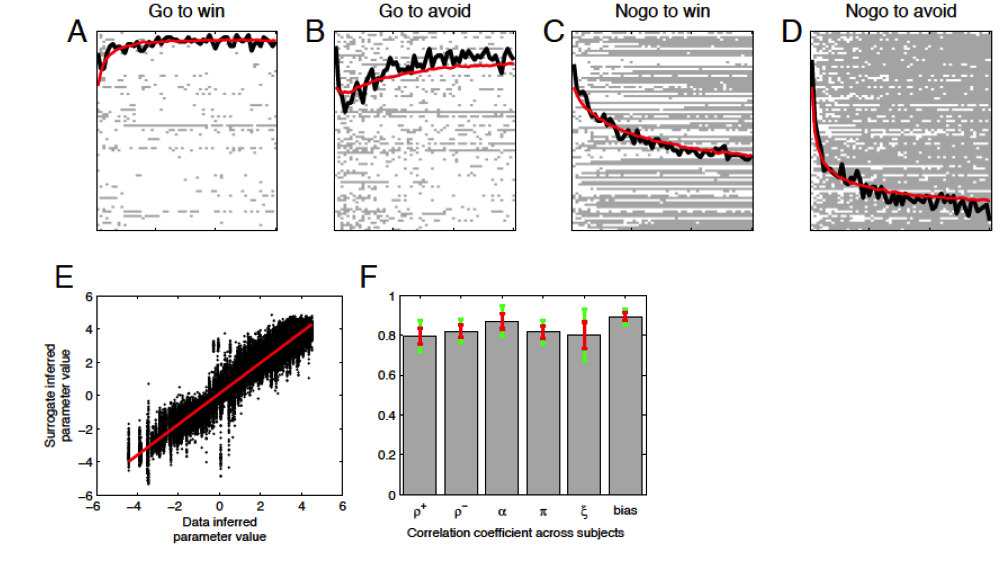
**

**Supplemental figure 2: testing the model selection with surrogated data**

We fitted the surrogate data using both the generative model (RW(rew/pun) +noise+bias+Pavovian) and the second best model that did not include any Pavlovian parameter (RW(rew/pun) +noise+bias). For each data set, we calculated the difference in iBIC (iBIC second best model – iBIC best model) and we found than in 99% of the datasets the winning model was the best model, the model used to generate the surrogate data.

**
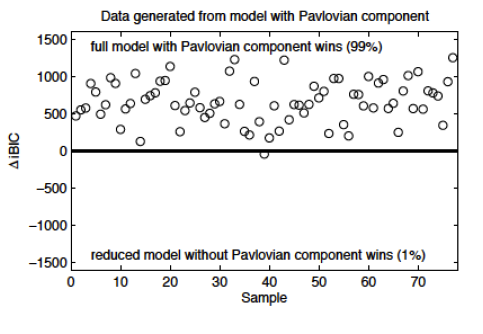
**

**Supplemental figure 3: learning curves for each four conditions**

The probability of a correct response was calculated for 6 consecutive bins of 10 trials each and displayed separately for each trial type in each treatment group. A mixed ANOVA with time bins, action (go/no-go) and valence (win/lose) as within subjects factors, and treatment (levodopa, citalopram, and placebo) as a between subjects factor revealed significant effect of time bin (F(2.9,242.3)=199.1, p<0.001), action by time bin (F(2.8,240)=10.5; p<0.001), and valence by time bin (F(3.3,280)=2.8; p=0.035), in the absence of action by valence by time bin, treatment by time bin, treatment by action by time bin, treatment by valence by time bin, or treatment by action by valence by time bin (all p>0.05). These learning curves show that the different treatment groups mainly differ in terms of the asymptote an not in terms of the initial learning slopes.
